# Biogeographical distribution and community assembly of Myxococcota in mangrove sediments

**DOI:** 10.1186/s40793-024-00593-2

**Published:** 2024-07-13

**Authors:** Dayu Zou, Cuijing Zhang, Yang Liu, Meng Li

**Affiliations:** 1 Archaeal Biology Center, Institute for Advanced Study, Shenzhen University, Shenzhen, 518060 China; 2 Institute for Advanced Study, Shenzhen Key Laboratory of Marine Microbiome Engineering, Shenzhen University, Shenzhen, 518060 China; 3 Synthetic Biology Research Center, Shenzhen University, Shenzhen, 518060 China

**Keywords:** Myxococcota, Mangroves, Biodiversity, Community assembly

## Abstract

**Background:**

Myxococcota, characterized by their distinct social lifestyles, are widely distributed micro-predators in global sediments. They can feed on a wide range of bacterial, archaeal, and fungal prey. Myxococcota are capable of producing diverse secondary metabolites, playing key roles in microbial food webs, and regulating the microbial community structures in different ecosystems. However, Myxococcota are rarely pure cultured due to the challenging and stringent culturing conditions. Their natural distribution, niche differentiation, and predator–prey relationships in a specific habitat are poorly understood.

**Results:**

In this study, we conducted a comprehensive analysis of the 16S rRNA gene sequence data from public databases and our collection. We compared the abundance, diversity, and distribution patterns of Myxococcota in various habitats, with a specific focus on mangroves. We found that Myxococcota accounted for 1.45% of the total prokaryotes in global sediments based on the abundance of 16S rRNA genes. Myxococcota are abundant and diverse in mangrove sediments. They tend to be more generalistic in mangroves than in other habitats due to their wide niche breadth. Besides, the deterministic processes (variable selection) influenced the assembly of mangrove Myxococcota communities significantly more than stochastic processes. Further, we determined that environmental factors explained a greater amount of total community variation in mangrove Myxococcota than geographical variables (latitude and sediment depth). In the end, through the analysis of microbial co-occurrence networks, Myxococcota emerges as a key component and functions as a connector in the mangrove microbial community.

**Conclusions:**

Our study enhances comprehension of mangrove Myxococcota's biogeography, assembly patterns, driving factors, and co-occurrence relationships, as well as highlights their unique niche and ecological importance in mangrove sediments.

**Supplementary Information:**

The online version contains supplementary material available at 10.1186/s40793-024-00593-2.

## Introduction

Prokaryotes dominate the Tree of Life, playing important roles in driving the bulk of global biogeochemical cycles, but our understanding of the processes generating their diversity and distribution is still limited. The “Myxobacteria” are characterized by their sophisticated multicellular lifestyle and were originally assigned to the class *Deltaproteobacteria* [1]. A recent phylogenomic assessment provides new evidence that supports the recognition of these organisms as a separate phylum (Myxococcota) based on their distinct metabolic and structural characteristics [2]. The highly social Myxococcota commonly exhibit specific behaviors regarding to predation and fruiting body formation. These gliding microorganisms can synthesize hundreds of carbon skeleton metabolites and derivatives, biodegrade many chemical compounds, and are important reservoirs of novel bioactive secondary metabolites and antibiotics [3–6]. Intriguingly, members of Myxococcota may have photosynthetic abilities and live a chimeric lifestyle in various nature environments [7]. As a result, they have a wide range of applications in agriculture, biomedicine, and environmental protection.

Despite much interest in their ability to synthesize useful metabolites and antibiotics, the natural distribution of Myxococcota remains poorly understood. Myxobacteria commonly grow slowly, do not readily form identifiable colonies on agar, and do not disperse well in liquid [4, 8, 9]. Accordingly, most myxobacteria are uncultured and uncharacterized due to the lack of optimal isolation methods, with only 74 described species to date [6, 10, 11]. Based on culture experiments, although some myxobacteria can grow in psychrophilic and thermophilic conditions, as well as in acidic or alkaline environments, most of them are mesophiles and neutrophiles [12–15]. Cultivation-independent techniques, such as high-throughput sequencing of 16S rRNA gene libraries, provide a more comprehensive and panoramic view of Myxococcota diversity and distribution: they are one of the most diverse bacterial groups and can be found almost everywhere in terrestrial and marine habitats [6, 11].

Mangrove wetlands are widely distributed along tropical and subtropical coastlines, serving as a link between the land and sea. Mangroves exhibit specific ecological features under the influence of terrestrial inputs and tidal effects, which contribute to a relatively higher abundance and diversity of microorganisms in mangrove ecosystems [16–18]. Although Myxococcota were suggested to be abundant and diverse in terrestrial and marine sediments [11], detailed information about the composition, assembly process, and distribution pattern of Myxococcota communities remains uncovered, especially in mangroves. Most importantly, myxobacteria may regulate local microbial communities, as these micropredators are able to prey on many other bacteria, archaea, and fungi [6, 19–21]. The predator–prey relationship is indispensable for the microbial food web, which may have further influences on the equilibrium and stability of ecosystems. Therefore, revealing the diversity and abundance of Myxococcota, analyzing their assembly and distribution patterns, as well as depicting their interactions with other microbes, is necessary to understand the niche differentiation and ecological importance of Myxococcota in mangrove sediments.

This study aims to describe the global distribution of sedimentary Myxococcota and compare their diversity and niche specificity in different sediment habitats on a large scale, with a focus on mangroves. Firstly, using publicly available 16S rRNA gene sequence dataset [16], we investigated the relative abundance and composition of the Myxococcota community in mangroves, rivers, freshwater lakes, coastal zones, oceans, saltwater lakes, and hot spring sediments. We analyzed the community assembly processes and reconstructed microbial co-occurrence networks for Myxococcota in diverse sediment environments. Secondly, we collected mangrove sediments in the south part of China to determine the major driving factors that influence the mangrove Myxococcota community. Finally, based on co-occurrence correlation, we explored potential predatory-prey interactions between Myxococcota and other bacteria and archaea in various environments. These analyses may enable us to gain a better understanding of the distribution pattern and unique niche of Myxococcota in mangrove sediments, which may help us explore the potential ecological roles of Myxococcota and emphasize their significance as key components of the mangrove microbial community.

## Materials and methods

### Sediment sampling, variable analysis, and 16S rRNA gene sequencing

Sediment samples were collected at Shenzhen Futian National Nature Reserve (SZ) in Southeast China. From February 2021 to January 2022, surface sediment samples (0–10 cm) were collected from three mangrove sites (MG1, MG2, MG3) and three mudflat sites (MF1, MF2, MF3) using a grab sampler during the dry season (October to March) and the wet season (April to September). To assess the vertical profiles of microbial communities, samples were collected in 2 cm depth intervals (from 0 to 30 cm) at MG1 and MF1 in August 2020, and in May, August, and November 2021, and January 2022 using a stainless-steel sampler. Based on the criteria of Luis et al. [22] and our sampling results, sediment samples were categorized into three depth layers, including the oxic layer (0 to 10 cm) and the anoxic layers (10 to 20 cm and 20 to 30 cm). In total 131 sediment samples were collected in this study (see supplementary table S3).

The geographical and environmental variables of each sample were summarized in Table S3. Geographical factors included the longitude, latitude, sampling depth, as well as temperature and mean annual precipitation (MAP) (obtained from the China Meteorological Administration [http://www.cma.gov.cn]). Environmental factors represented the physicochemical properties of each sediment sample, including salinity, pH, total carbon (TC), total organic carbon (TOC), total nitrogen (TN), ammonium (N/NH_4_^+^), nitrate (N/NO_3_^−^), total phosphorus (TP), and total sulfur (TS), and were determined as described previously [16].

The PowerMax soil kit (Qiagen, Germany) was used according to the manufacturer's instructions to extract DNA from 0.5 g of wet sediments in triplicate for each sample. Triplicates of each DNA sample were combined and well-mixed prior to sequencing and analysis. Using the primer pairs 515F/806R recommended in the Earth Microbiome Project (EMP) [23], prokaryotic (Bacteria and Archaea) 16S rRNA gene fragments were amplified and sequenced on the HiSeq platform with a 250-bp pair-end strategy (Illumina, San Diego, CA, USA) at Magigene (Shenzhen, China).

### Data collection and processing

To compare Myxococcota community between mangrove sediment and other biomes (i.e., the freshwater river, freshwater lake, coastal zone, ocean, saltwater lake, and hot spring environments), we utilized the 16S rRNA gene dataset (i.e., the sequencing data on sediment prokaryotic diversity using 515F/806R primers from published data and EMP datasets) described in our previous study [16]. This dataset also contained the sequencing data of other mangroves in China, including Ximendao National Marine Reserve (XMD), Yunxiao Zhangjiangkou National Nature Reserve (YX), Leizhou Nature Reserve (LZ), Dongzhaigang National Nature Reserve (DZG), and Danzhou Xinyinggang Nature Reserve (DZ). Overall, 1191 published sediment amplicon sequencing data from 200 sampling sites were used in this study (Table S1). All the OTU table of each data set were merged using “qiime feature-table merge” in the QIIME2 platform (v 2022.2) [24]. To minimize sequencing depth bias, the resultant OTU table was rarefied to 10,000 sequences per sample as previously described [16]. Myxococcotal OTUs were retrieved, their presence and absence in different biomes were revealed as well. Alpha diversity indices, including OTU richness and Shannon–Wiener indices of the Myxococcota community, were calculated using “qiime diversity alpha”.

Raw data obtained in the current study were processed using the QIIME2 pipeline. Firstly, sequencing data were imported into the QIIME2 platform using “qiime tools import”, the quality filtering was employed by “qiime quality-filter q-score” with the setting as p-min-quality 20 and p-min-length-fraction 0.85. Secondly, the merging and denoising of paired reads, chimera removal, and feature generation were employed by DADA2 using “qiime dada2 denoise-paired” with the p-min-overlap as 12 [25]. Features were clustered at 97% sequence identity using “qiime vsearch cluster-features-de-novo” in the vsearch module to align the representative OTUs. Taxonomic information for each representative OTUs was assigned in the feature-classifier module using “qiime feature-classifier classify-sklearn” according to the SILVA Nr99 database (v138) [26].

### Statistical analysis

Comparisons of the Myxococcota community assembly patterns and niche differentiations in diverse habitats

Te 1191 published sample set of different biomes was used in the below analyses (Table S1). The Levins' niche breadth of the Myxococcota community in different habitats was calculated by the R script “niche.width” in the R package spaa (https://github.com/helixcn/spaa). Analyses of variance (ANOVA) were employed to compare the alpha diversity of major prokaryotic phyla across different habitats. The relative abundance and the niche breadth of the Myxococcota in different biomes were compared as well. Significant differences (*p* < 0.05) among groups were evaluated using the analysis of similarities (ANOSIM). Principal coordinate analysis (PCoA) and permutational multivariate analysis of variance (PERMANOVA) were used to depict the community composition shift of total prokaryotic and Myxococcota among different biomes based on the Bray–Curtis dissimilarity matrices on the genus level. The above analyses were conducted using the vegdist and anosim function in the vegan package in R (version 3.6, R Development Core Team, Vienna, Austria).

The neutral community model (NCM) was employed to compare the potential roles of stochastic processes in shaping Myxococcota community assembly in different biomes by predicting the relationship between occurrence frequency and relative abundance of Myxococcota OTUs. The fitness to the NCM was evaluate using the R script sncm.fit_function.r, which was written by Burns et al. and attached as a supplementary code of their paper [27]. The beta nearest-taxon index (βNTI) was used to verify the relative importance of stochastic and deterministic processes of the Myxococcota community. Briefly, βNTI is the number of standard deviations of the beta mean nearest taxon distance from the mean of the null distribution [28]. |βNTI|< 2 suggests stochastic processes may be dominant in microbial community assembly, |βNTI|> 2 indicates deterministic processes (homogeneous selection and heterogeneous selection) may play more important roles in community assembly than stochastic processes [28, 29]. The Bray–Curtis-based Raup-Crick (RC_bray_) value, characterized the magnitude of deviation between observed Bray–Curtis (BC_obs_) and Bray–Curtis expected under the randomization (BC_null_), was used to further partition pairwise comparisons that were assigned to stochastic processes (i.e., when |βNTI|< 2) [30]. In this case, |RC_bray_|< 0.95 suggests the undominated processes for community assembly, while RC_bray_ < -0.95 and RC_bray_ > 0.95 indicates the crucial influence of homogenizing dispersal and dispersal limitation, respectively [30]. The percent of mangrove Myxococcota community assembly governed primarily by various deterministic and stochastic processes was calculated based on the above-mentioned methods. The null model analyses were conducted using the “cal_ses_betamntd” and “cal_rcbray” in the “trans_nullmodel” function of the R package microeco followed by the instructions (https://rdrr.io/github/ChiLiubio/microeco/man/trans_nullmodel.html) [31]. Linear discriminant analysis effect size (LDA effect size, LEfSe) was used to determine the enrichment for certain Myxococcota groups in saline or non-saline habitats on the genus level, with the α-value for the factorial Kruskal–Wallis test as 0.05 and the threshold for the logarithmic LDA score as 2.0. LEfSe analyses were performed with a web-based bioinformatics tools implemented in MicrobiomeAnalyst 2.0 [32].

To reveal and compare the potential interactions between Myxococcota and other prokaryotes in different biomes, co-occurrence networks were built based on the abundance profiles of individual OTUs. OTUs with a relative abundance fraction larger than 0.01% and present in at least half of the samples for each biome were included. As a result, 456, 549, 429, 476, 410, 526, and 213 OTUs were used in constructing the network in mangrove, coastal, marine, salt-lake, freshwater lake, river, and hot spring. Microbial interactions with the Spearman’s coefficient |R|> 0.7 and *p* < 0.01 were retained. The random matrix theory (RMT)-based approach was used for network construction, topological roles identification, module membership with an automatic threshold using iNAP online platform [33]. To characterize the modularity property, each network was separated into modules by the fast greedy modularity optimization. The basic network topological properties were calculated, including numbers of nodes, edges, and modules, R square, modularity, average degree (avgK), and average path distance (GD). The betweenness centrality (BC), revealing the role of nodes as a bridge between network components, was compared in different biomes of Myxococcota nodes. The among-module connectivity (*P*_*i*_) and within-module connectivity (*Z*_*i*_) of Myxococcota nodes were also compared in different biomes, which categorizes the different topological roles of nodes as peripherals (*Z*_*i*_ ≤ 2.5, *P*_*i*_ ≤ 0.62), connectors (*Z*_*i*_ ≤ 2.5, *P*_*i*_ > 0.62), module hubs (*Z*_*i*_ > 2.5, *P*_*i*_ ≤ 0.62), and network hubs (*Z*_*i*_ > 2.5, *P*_*i*_ > 0.62) [34]. Networks were visualized through Cytoscape (version 3.7.2).

Exploring spatial variations and vertical distribution patterns of Myxococcota in mangrove ecosystems

The detailed community composition of Myxococcota in 185 Chinese mangrove samples was revealed at the family level and used in the analyses subsequently (Table S3). The relative abundance of Myxococcota 16S rRNA gene in different mangroves was compared, and the significant differences (*p* < 0.05) were calculated using ANOSIM. PCoA and PERMANOVA based on the Bray–Curtis dissimilarity matrices on the genus level were performed to test whether prokaryotic community compositions shifted among different biomes.

The Bray–Curtis based PERMANOVA was employed to test whether the Myxococcota community compositions (in OTU level) varied between different sample groups, which was visualized using PCoA. Variance inflation factor (VIF) was used to verify the linearity relationship between all factors, and to select non-linear geographical (i.e., latitude, depth, temperature, and MAP) and environmental factors (i.e., salinity, pH, TOC, TS, TP, TN, N/NH_4_^+^, and N/NO_3_^−^) for further analysis. The significance of the associations between environmental factors and the abundance, diversity, and community composition of Myxococcota were assessed using Mantel tests with Pearson’s correlation coefficient. The result of detrended correspondence analysis (DCA) suggested that RDA was better to depict the influence of factors on the ordination of Myxococcota communities in samples based on the Bray–Curtis’s distance (db-RDA). Variation partition analysis (VPA) based on redundancy analysis (RDA) was performed to determine the relative proportions of community variation that could be explained by geographical and environmental variables combined. The above statistical analyses were performed using vif.cca and ordiR2step function in the vegan package in R (version 3.6, R Development Core Team, Vienna, Austria).

## Results

### The diversity, composition, and assembly patterns of Myxococcota communities in different biomes

**Fig. 1:**
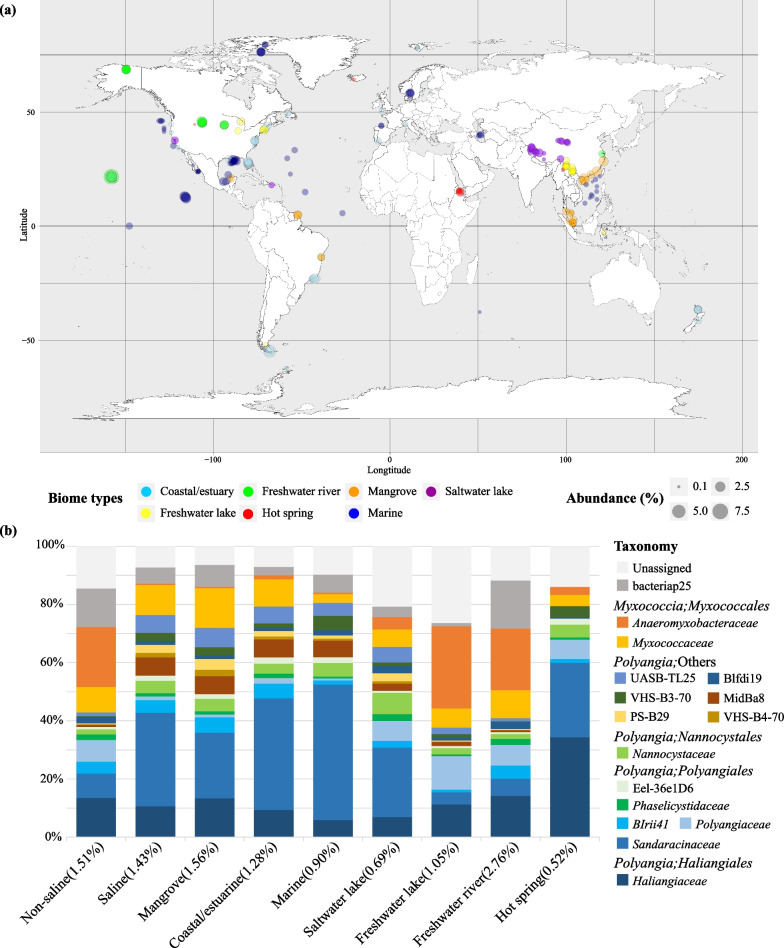
Global distribution and relative abundance of Myxococcota in sediments (**a**). The family-level composition of sediment Myxococcota community in different habitats (**b**). Numbers in parentheses indicate the average abundance fraction of myxococcotal 16S rRNA genes in each biome

**Fig. 2:**
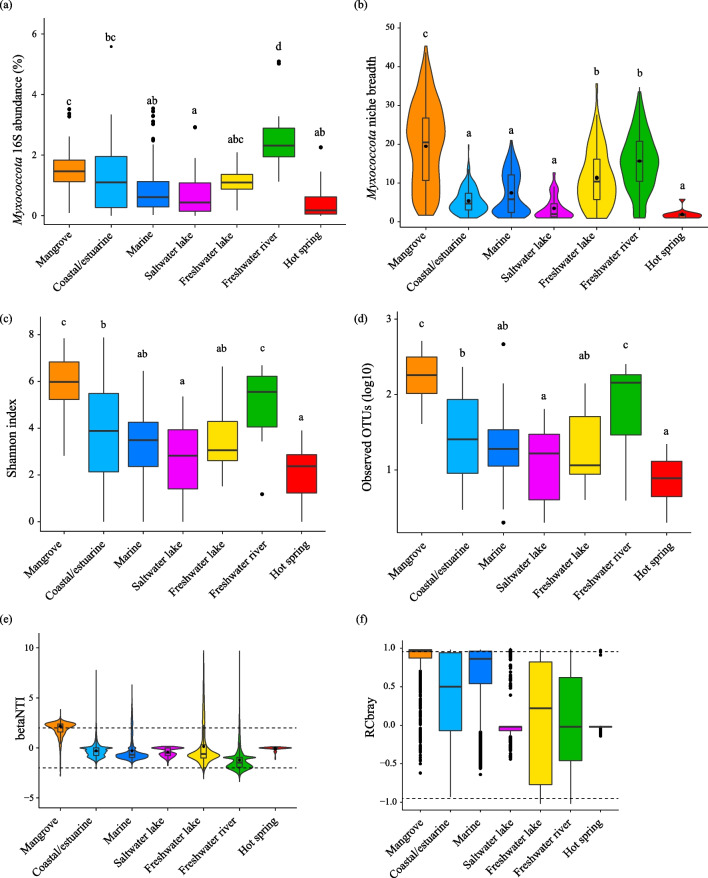
The relative abundance (**a**), niche breadth (**b**), Shannon index (**c**) and OTUs richness (**d**) of Myxococcota in different biomes. Beta-nearest-taxon index (βNTI) of the Myxococcota communities in different environments (**e**). Horizontal dashed lines (βNTI values at 2 and − 2), thresholds of significance. Bray–Curtis-based Raup-Crick (RC_bray_) values of the Myxococcota communities in different environments (**f**). Horizontal dashed lines, RC_bray_ values at 0.95 and − 0.95. Black dots represent outliers. Different letters indicate significant differences among different biomes (*p* < 0.05)

Myxococcotal 16S rRNA genes were observed in various environments (Fig. 1a). Compared to other commonly observed prokaryotic predators, such as Bdellovibrionota (mean abundance fraction 0.32%), Myxococcota showed higher abundance (mean abundance fraction 1.45%, *p* < 0.05) in all sediment samples. Although there was no significant difference in the abundance of Myxococcota between saline (1.43%) and non-saline (1.51%) habitats (Table S1), it varied across different biomes, ranging from 0.52% in hot springs to 2.76% in freshwater rivers (Fig. 1b). The relative abundance of Myxococcota ranked highest in mangroves (1.56%) among saline habitats, followed by coastal/estuarine (1.28%), marine (0.90%), and salt lakes (0.69%). (Fig. 1b and Fig. 2a). At the family level, Anaeromyxobacteraceae, Polyangiaceae, and Haliangiaceae were predominant in non-saline environments, while UASB-TL25, MidBa8, PS-B29, VHS-B4-70, Nannocystaceae, and Sandaracinaceae were enriched in saline biomes (Fig. 1b). The LEfSe results revealed the salinity preference of Myxococcota members in this study (Fig. S2). Sandaracinaceae, MidBa8, Nannocystaceae*,* and VHS_B4_70 were suggested as biomarkers for saline environments, while the biomarkers in non-saline habitats were Anaeromyxobacteraceae, Haliangiaceae, Polyangiaceae*,* 27F_1492R, Phaselicystidaceae, and mle1_27.

**Fig. 3:**
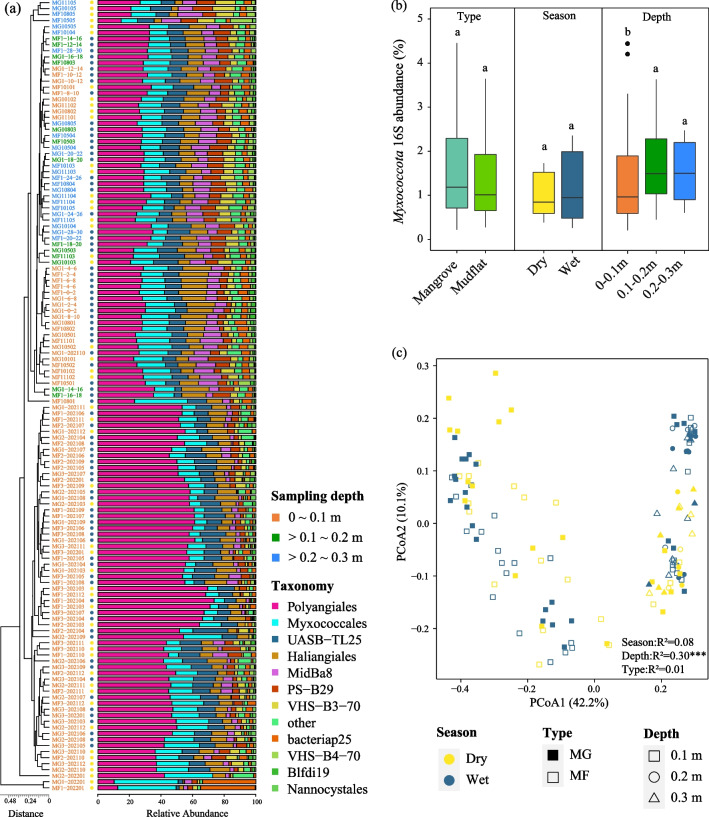
The family-level composition and Bray–Curtis-based clustering of mangrove Myxococcota community (**a**). Colors of the sample name represent different sampling depths. Yellow and blue dots behind the sample name represent dry and wet seasons. The comparison of mangrove Myxococcota 16S gene abundance of samples collected in mangroves (MG) and mudflats (MF), wet and dry seasons, and different sampling depths (**b**). Black dots represent outliers. Different letters indicate significant differences among different groups (ANOVA, *p* < 0.05). The PCoA result of Myxococcota community in samples collected in different seasons, depths, and types (i.e., MG and MF) based on the Bray–Curtis distance matrix (c). Similarity values among the samples of different seasons (“Season”), depths (“Depth”), and types (“Type”) were examined by using the analysis of similarities (ANOSIM) and are shown in the bottom right or left corner of this graph. ***p < 0.001

Myxococcota exhibited a wider range of niche breadth value in mangroves than that in other biomes (Fig. 2b). The Myxococcota community exhibited significantly (*p* < 0.05) higher Shannon index and OTU richness in mangroves compared to other saline habitats (Fig. 2c and 2d). The PCoA results displayed differences in beta diversity of the total microbial community and the Myxococcota community between mangroves and other non-saline environments (Fig. S3a and S3b). The PERMANOVA based on Bray–Curtis dissimilarity confirmed that biome types had a significant impact on the composition of the Myxococcota community (*p* = 0.001). In total 1832 myxococcotal OTUs were obtained in this study (Table S2). There were 334 myxococcotal OTUs in both saline and non-saline environments, while 1218 and 280 OTUs were uniquely observed in saline and non-saline environments, respectively (Fig. S3c). Among the 766 myxococcotal OTUs identified in mangroves, 64.5% of the myxococcotal OTUs were observed in other saline habitats, mainly from the coastal and estuarine environment (Fig. S3d). However, only 6.5% of mangrove myxococcotal OTUs were shared between mangroves and non-saline environments (Fig. 3c), with the majority originated from rivers (Fig. S3e). Besides, 28.9% (222 OTUs) of myxococcotal OTUs were only presented in mangroves, which mainly consisted of Myxococcaceae (25%), Sandaracinaceae (14%), Haliangiaceae (9%), and Blfdi19 (6%) groups (Fig. S3f).

Among all biomes, the NCM fits well with Myxococcota community assembly in mangrove and marine environments (R^2^ > 0.6) (Fig. S4). Since the NCM did not explain the entire assembly process of the Myxococcota community in mangroves, the βNTI and RC_bray_ were utilized to further investigate the impact of stochastic and deterministic processes on the assembly of the Myxococcota community. Clearly, the majority (65.5%) of βNTI values were greater than 2 in mangroves, which highlighted the more important role of deterministic processes (variable selection) in assembling the Myxococcota community compared to other habitats (Fig. 2e). Most of RC_bray_ values (75.8%) were larger than 0.95, suggesting a more crucial role of dispersal limitation in the assembly of Myxococcota community than that of homogenizing dispersal and undominated processes (Fig. 2f). Considering all the above influencing processes, variable selection (50.5%), followed by dispersal limitation (28.6%), was the most contributing process controlling the Myxococcota community assembly in mangroves.

### Geographical and environmental selection shaping the Myxococcota community in mangroves of China

The community composition and abundance of Myxococcota varied between different mangroves in China (Fig. S5a and Fig. S5b). The PCoA results showed that the location of the mangroves had a significant influence (PERMANOVA, *p* = 0.001) on the Myxococcota community (Fig. S5c). For samples collected in the Shenzhen Futian National Nature Reserve (MG and MF sites), the most abundant myxococcotal order was Polyangiales (38.1%), followed by Myxococcales (16.3%) and Haliangiales (11.1%) (Fig. 3a). In terms of family level, Sandaracinaceae (28.8%), Myxococcaceae (11.2%), and Haliangiaceae (11.1%) collectively accounted for more than half of the Myxococcota community. In Chinese mangroves, the Myxococcota community was primarily divided and clustered based on sampling depth (Fig. 3a and Fig. 3c). However, the sample types (mangrove or mudflat) and sampling seasons (wet or dry) had no significant influences on the abundance and community composition of Myxococcota.

**Fig. 4:**
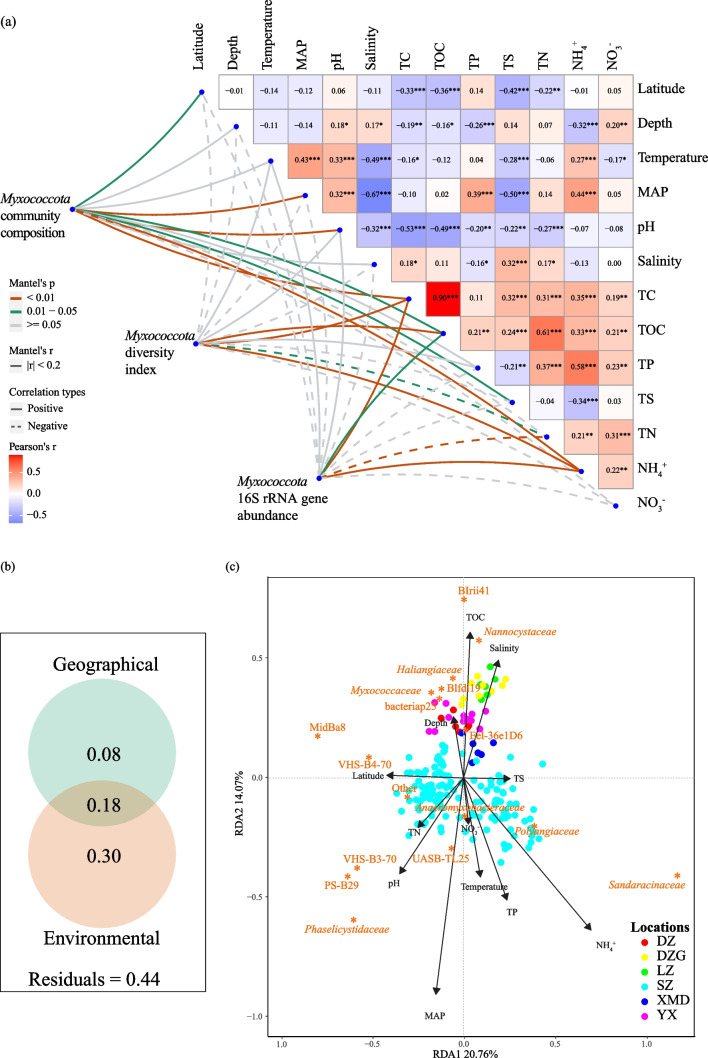
Mantel tests showing the Pearson’s correlations between Myxococcota community composition, diversity index, 16S rRNA gene abundance, and environmental and geographical factors (**a**). Significance: *, *p* < 0.05; **, *p* < 0.01; ***, *p* < 0.001. Variation partition analysis based on Bray–Curtis dissimilarity matrices, partitioning the relative contributions of geographical and environmental factors to Myxococcota community structure in mangrove (**b**). The results of distance-based redundancy analysis (db-RDA), exhibiting the effects of variables on the Myxococcota communities in family level. Different colored circles represent the different location of samples (**c**)

The Pearson’s correlation revealed strong connections between latitude and depth with other environmental factors (*p* < 0.05), indicating significant differences in sediment physicochemical parameters among different mangroves (Fig. 4a). According to the Mantel test results, the composition and abundance of the Myxococcota community was mainly influenced by TC, TOC, NH_4_^+^. The VPA revealed that 56.0% of the variations in the Myxococcota community were explained by geographical and environmental factors, with a greater contribution from environmental factors (Fig. 4b). Further, RDA analysis revealed that the first two axes accounted for 34.8% of the total variance. It was found that MAP, TOC, and NH_4_^+^ were the most influential variables impacting the compositions of the Myxococcota community.

### Co-occurrence patterns and niche differentiation of Myxococcota community in mangroves

**Fig. 5:**
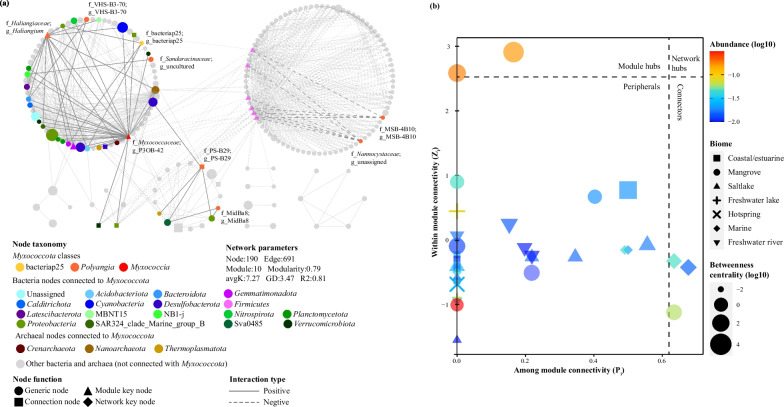
Co-occurrence networks of Myxococcota communities in all mangrove samples (**a**). Nodes of bacteriap25, Polyangia, and Myxococcia are colored by yellow, orange, and red, respectively. Other archaeal and bacterial nodes that are associated with Myxococcota nodes are colored according to the phylum. Edges connecting Myxococcota nodes with other nodes are thick and black. Node size is proportional to the relative abundance of each node. avgK, average degree; GD, average path distance. The roles of Myxococcota nodes in the microbial co-occurrence networks of different biomes (**b**). Colors and shapes stand for the relative abundance and original habitat of each node. Symbol size is proportional to the betweenness centrality of each node

Based on Spearman’s correlation, co-occurrence networks were generated for the overall prokaryotic community in mangroves (Fig. 5a) and other biomes (Fig. S6). The network constructed in the mangrove environment exhibited the highest modularity compared to all other networks, which consisted of 190 nodes, 691 edges, and 10 modules (Fig. 5a). The Myxococcota nodes observed in the co-occurrence network were mainly affiliated with bacteriap25, Polyangia, and Myxococcia. Compared to other biomes, the mangrove network contained a greater variety of Myxococcota groups, with 8 different families. Similarly, the connections related to Myxococcota and other prokaryotic nodes were more complex, with 58 edges. These connections encompassed 16 bacterial phyla and 3 archaeal phyla. Similarly, mangrove Myxococcota nodes showed higher betweenness centrality (BC) values, indicating their broader roles in connecting different network components. Further, Myxococcota may function as module hubs and connectors in mangroves, as indicated by the *P*_*i*_ and *Z*_*i*_ values of myxococcotal nodes (Fig. 5b).

## Discussion

### Salinity drives the global distribution and differentiation of Myxococcota

It is commonly noted that there are phylogenetic divergences between microbial communities from freshwater and marine habitats. Myxococcota were thought to exclusively dwell in terrestrial habitats for a long time, until species were isolated from beach sand [35], estuarine and coastal salt marshes [36, 37], and other marine habitats [38, 39]. Subsequently, several Myxococcota isolates were obtained from saline-alkaline soils [40], further confirming that some of them were halotolerant or halophilic. A recent study claimed that myxobacteria are one of the most diverse bacterial groups on Earth, accounting for 2.34% of the total bacterial OTUs [11]. Although Myxococcota were detected globally in both free-living and host-associated environments, they showed a preference for non-saline soil environments [11]. Here, we determined that Myxococcota accounted for 1.45% of the total prokaryotes in global sediments, while the relative abundance of Myxococcota showed no significant differences between saline and non-saline sediments (*p* < 0.05, Fig. S1b). This finding further suggests that Myxococcota may be a cosmopolitan and key component of the benthic microbial community on Earth. Besides, we identified the salinity preference of Myxococcota groups (Fig. S2 and S3c), and also observed clear differences in the community composition between saline and non-saline habitats (Fig. 1b). Clades MidBa8, VHS_B4_70, and UASB-TL25 were reportedly enriched in saline biomes, and both Sandaracinaceae and Nannocystaceae had a high incidence and relative abundance in saline environments [6, 11]. As for groups predominant in non-saline environments, sequences of Polyangiaceae were rarely detected in saline biomes, implying that they may not adapt to saline habitats. Anaeromyxobacteraceae were found to be abundant in low-salinity lake, pond, and wetland sediments. Clades Phaselicystidaceae, Bacteriap25, and Mle1_27 were commonly distributed in terrestrial soils and freshwater sediments [6, 11]. The Bray–Curtis based PERMANOVA and PCoA results also demonstrated clear community variations between different biomes (Fig. S3a and S3b). Therefore, our findings underline the divergent salinity preferences among different Myxococcota groups, which also implies the crucial influence of salinity on the composition and distribution of Myxococcota.

### Environmental selection plays an important role in shaping the Myxococcota community in mangrove sediments

Due to river runoff and tidal effects, mangrove sediments always exhibit unique environmental properties, such as a high level of nutrients, changing salinity and oxygen conditions, and diverse organic compounds [41, 42]. Notably, Myxococcota dwelling in mangrove sediments showed the widest niche breadth compared to other environments (Fig. 2b). Niche breadth refers to the range of resources that a species uses, reflecting the tradeoff of evolutionary processes on resource utility and stress tolerance, and further implies the environmental adaptations by a single species or a population. It also determines the geographical distribution of a species [43–45]. Among different biomes, the wide niche breadth of Myxococcota suggested that they were more inclined towards being generalists in mangroves. This may also indicate their broad distribution and wide range of resource utilization to adapt to the nutrient-rich but fragile mangrove ecosystem. A recent study revealed that the mangrove myxobacterial community had diverse metabolic potentials and was an important reservoir of novel secondary metabolite compounds [46]. These characteristics can be attributed to the strong adaptability and plasticity of Myxococcota in response to environmental disturbances, particularly in the richly diverse mangrove sediments.

Previous studies have observed the high fitness of NCM for the mangrove microbial community, which demonstrates the potential roles of stochastic processes in community assembly [16, 17]. Similarly, stochastic processes also had influences on the Myxococcota community assembly in mangroves (Fig. S4). Besides, the high immigration rates may suggest the invasion of various marine groups, resulting in a mixture of saline and non-saline groups in mangroves. This is consistent with our observation that coastal and river biomes may be the major source of mangrove myxococcotal OTUs, which indicated the influence of tidal effects and river runoff on the mangrove Myxococcota community. According to the βNTI and RC_bray_, variable selection and dispersal limitation were the most influential processes in the assembly of the mangrove Myxococcota community (Fig. 2e and 2f). This highlights the significance of local environmental factors in driving the assembly of the Myxococcota community.

To further explore the abundance, diversity, and drivers of mangrove Myxococcota_*,*_ we compared the Myxococcota community in representative mangroves of China. We observed significant variations in the community structure of Myxococcota among different mangroves (ANOSIM, *p* < 0.001, Fig. S5) and in different sampling depths (ANOSIM, *p* < 0.001, Fig. 3). Seasonal variations of microbial communities in sediments are widely reported in various environments [47–49]. A recent study suggested that Myxococcota played an essential role in shaping the microbial communities in alpine environments [50], and were responsible for the seasonal dynamics of the soil microbial food web mainly contributed by biotic interactions. However, we did not observe clear community variations of the Myxococcota between dry and wet seasons, and the differences were not significant between mangrove and mudflat samples (Fig. 3). This may indicate that the seasonal dynamics of Myxococcota communities vary among different biomes. Mangrove Myxococcota communities in China are almost stable with changing seasons, suggesting their potential adaptions to the locale environment.

The VPA provided strong evidence that environmental factors had a stronger influence on Myxococcota diversity and composition than latitude and sediment depth in this study (Fig. 4b). Myxococcota may exhibit distinct behaviors and encode different metabolic pathways in oxic or anoxic sediments [51]. The aerobic dwellers may possess a highly sophisticated machinery functioned in predation and cellular differentiation behaviors. However, some Myxococcota were predicted to be strict anaerobes and lack the capacity for predation and social differentiation, while they may gain energy through utilizing fermentation, nitrate reduction, and dissimilarity sulfate reduction. Besides, the observation of some chlorophotrophic Myxococcota in surface sediments also suggested that depth was one crucial factor differentiating the composition and function of Myxococcota communities [7]. However, the limited sampling depth in the current study may be not deep enough to exhibit the vertical distribution patterns in mangroves. Moreover, considering the active gliding of Myxococcota and the effect of vertical movement by other organisms in sediments it is necessary to evaluate the influence of depth on Myxococcota communities, which may need further explorations. Similar findings were previously reported in other soil and sediment ecosystems [52, 53], which emphasizes the influence of local environmental conditions on the microbial distribution. As additional evidence, environmental factors such as TC, TOC, and NH_4_^+^ were found to have a strong correlation with the abundance, diversity, and community structure of Myxococcota (Fig. 4a). TOC and NH_4_^+^ were reported as the most important factors affecting the myxobacterial community structure in soils [19, 20]. Besides, a wide range of carbohydrate-active enzymes (CAZymes) and peptidases have been discovered in various myxobacterial genomes [54, 55]. This finding could potentially explain the close relationship between the Myxococcota community and the organic compounds found in mangrove sediments. Further, the db-RDA revealed significant relationships between environmental factors and the relative abundances of Myxococcota families (Fig. 4c), suggesting that the influence of environmental factors was niche-specific. As such, the above observations have provided insights into the distribution and biogeography of Myxococcota in mangroves and highlight the crucial role of environmental factors in driving the community structure of Myxococcota.

### Myxococcota are key components that bridge the microbial co-occurrence network in mangrove sediments

Naturally, microorganisms prefer to form complex interaction networks in various ecosystems rather than thrive alone. Microbial interactions, such as antagonism or cooperation, are crucial components that affect the composition and dynamics of microbial communities [56]. Modularity reflects the stability and resilience of an ecosystem [57]. In the present study, the co-occurrence network of mangrove microbes exhibited the highest modularity value compared to all other networks (Fig. 5a and S6). This indicates that microbes preferentially work in groups, and microbial function modules are clearly divided in mangroves [58]. Notably, the mangrove co-occurrence network showed a relatively higher average path distance (GD) and a comparatively lower average degree (avgK) value, further proving that microbial interactions in mangrove habitats were much more complex and interconnected.

Different microbes may exhibit distinct topological roles in the microbial network as a result of niche variations and different ecological roles, which may also reflect their potential for environmental adaptations and unique distribution patterns [34, 59]. Most microbial nodes naturally have limited connections only within their own modules, which are known as peripherals. Here, certain groups of Myxococcota (i.e., *Haliangium* and *P3OB-42*) were identified as key module hubs in mangroves based on the values of P_i_ and Z_i_, but not in other environments (Fig. 5b). *Haliangium*, reported as a typical marine representative [60], is one of the most abundant genera of myxobacteria, which has potential roles in denitrification and phosphate solubilization [11, 61, 62]. Based on a recent large-scale geographic survey conducted in typical farmland soils, *Haliangium* was found to be crucial in predicting the soil multi-nutrient cycling index [63]. Similarly, *P3OB-42* is widely distributed and frequently detected in many habitats, where it plays a role in nitrogen and phosphate cycling [64]. Stable Isotope probing (SIP) experiments demonstrated that *P3OB-42* was a potential aerobic methanotroph in rice fields [65]. Although the genus *PS-B29* was rarely detected and reported, a few studies have observed its enrichment in high sulfide and methane-rich environments, suggesting its potential for sulfate reduction and methane oxidation [66, 67]. The genus *PS-B29*, served as the crucial connector between modules (Fig. 5a), which indicated their importance in mangrove sediments. Besides, the high BC values of these nodes also underline their importance as necessary intermediate transfers within major modules of the mangrove microbial network.

Myxococcota are one of the most important keystone components in the soil food web [68]. Previous studies have observed correlations between Myxococcota and other bacteria through the microbial co-occurrence network, which may imply potential predator–prey relationships [21, 63]. Dai et al. reported that myxobacteria may affect soil nutrient cycling, possibly due to their extensive predation on functional bacteria related to nutrient cycling [69]. We observed negative correlations between Myxococcota and Firmicutes (Fig. 5a), suggesting a potential predatory-prey relationship between them. However, on the contrary, there is limited information depicting the relationship between Myxococcota and archaea. *Myxococcus xanthus* is able to consume a broad range of microbial species, including archaea [70]. In this study, the positive correlations among Myxococcota and archaea groups may imply possible interactions in nutrient metabolism and electron transferring, which needs further verification and exploration. Thus, our work provides clues for further co-culture and microcosmic experiments along with studies to improve Myxococcota culturability, also sheds light on the uniqueness and irreplaceability of the diverse and abundant Myxococcota in mangroves.

## Conclusions

In conclusion, we found that salinity influenced the global distribution and differentiation of the Myxococcota community. Compared to other benthic ecosystems, mangrove Myxococcota exhibited higher diversity and abundance and wider niche breadth value, implying that they may be generalists with a wide range of food sources and stress tolerance. Environmental selection was more important in the community assembly of mangrove Myxococcota than other processes. Furthermore, environmental factors explained a higher proportion of community variation than geographical variables, while MAP, TOC, and NH_4_^+^ were the most important factors. Finally, Myxococcota functioned as keystone taxa that bridged different microbial modules in the co-occurrence network. The potential predator–prey relationships between Myxococcota and diverse bacterial and archaeal groups may suggest their influence on nutrient cycles in mangrove sediments. In summary, our findings provide new insights into the assembly patterns, differentiation driving factors, and ecological roles of Myxococcota in mangrove ecosystems.

### Supplementary Information

Additional file1 (PDF 2704 KB)

Additional file2 (XLSX 249 KB)

## Data Availability

The raw HiSeq sequencing data for 16S rRNA gene libraries in this study were deposited in the National Omics Data Encyclopedia (NODE) database with the BioProject accession number OEP004344.
