# Supplementary material for: Biogeographical distribution and community assembly of Myxococcota in mangrove sediments
Source: Environ Microbiome. 2024 Jul 13;19:47. doi: 10.1186/s40793-024-00593-2 (PMC11245791; doi:10.1186/s40793-024-00593-2)
Supplement: Supplementary file 1 — Additional file1 (PDF 2704 KB) [file 40793_2024_593_MOESM1_ESM.pdf]

Supplementary information

Biogeographical Distribution and Community Assembly of *Myxococcota* in Mangrove Sediments

Dayu Zou[1,2], Cuijing Zhang[1,2], Yang Liu[1,2*], Meng Li[1,2*]

[1]Archaeal Biology Center, Institute for Advanced Study, Shenzhen University, Shenzhen 518060, China

[2] Shenzhen Key Laboratory of Marine Microbiome Engineering, Institute for Advanced Study, Shenzhen University, Shenzhen 518060, China

*Corresponding author

Meng Li

email: limeng848@szu.edu.cn

Yang Liu

email: yangliu@szu.edu.cn

Supplementary figures

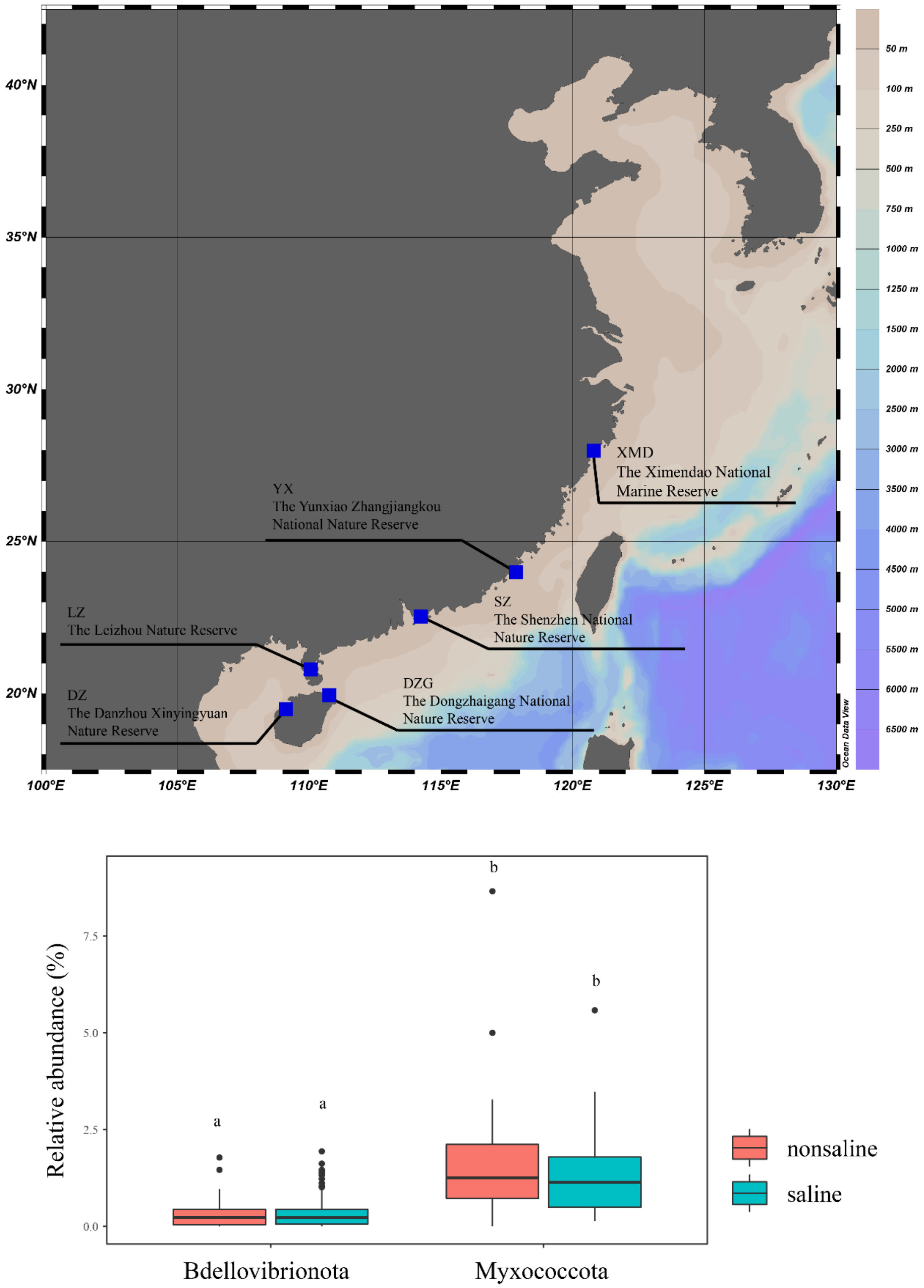

Figure S1 The location of mangrove sites (top). The relative abundance of *Myxococcota* and *Bdellovibrionota* in saline and non-saline environments (bottom). Different letters indicate significant differences among different groups (ANOVA, $p < 0.05$).

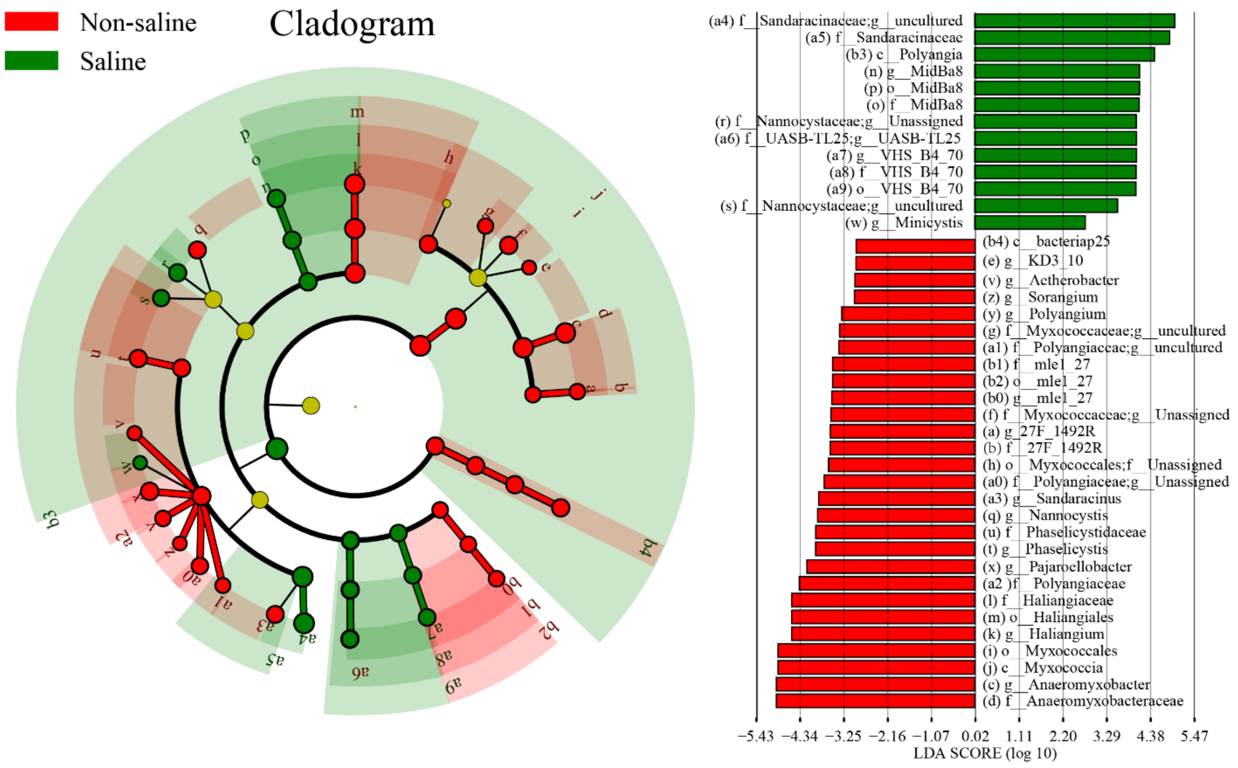

Figure S2 *Myxococcota* taxa significantly differentiated between the saline and non-saline sediments identified by linear discriminant analysis coupled with effect size (LEfSe). Lowercase letters in parentheses represent their location in the cladogram. Taxonomic units are abbreviated as c (class), o (order), f (family), and g (genus).

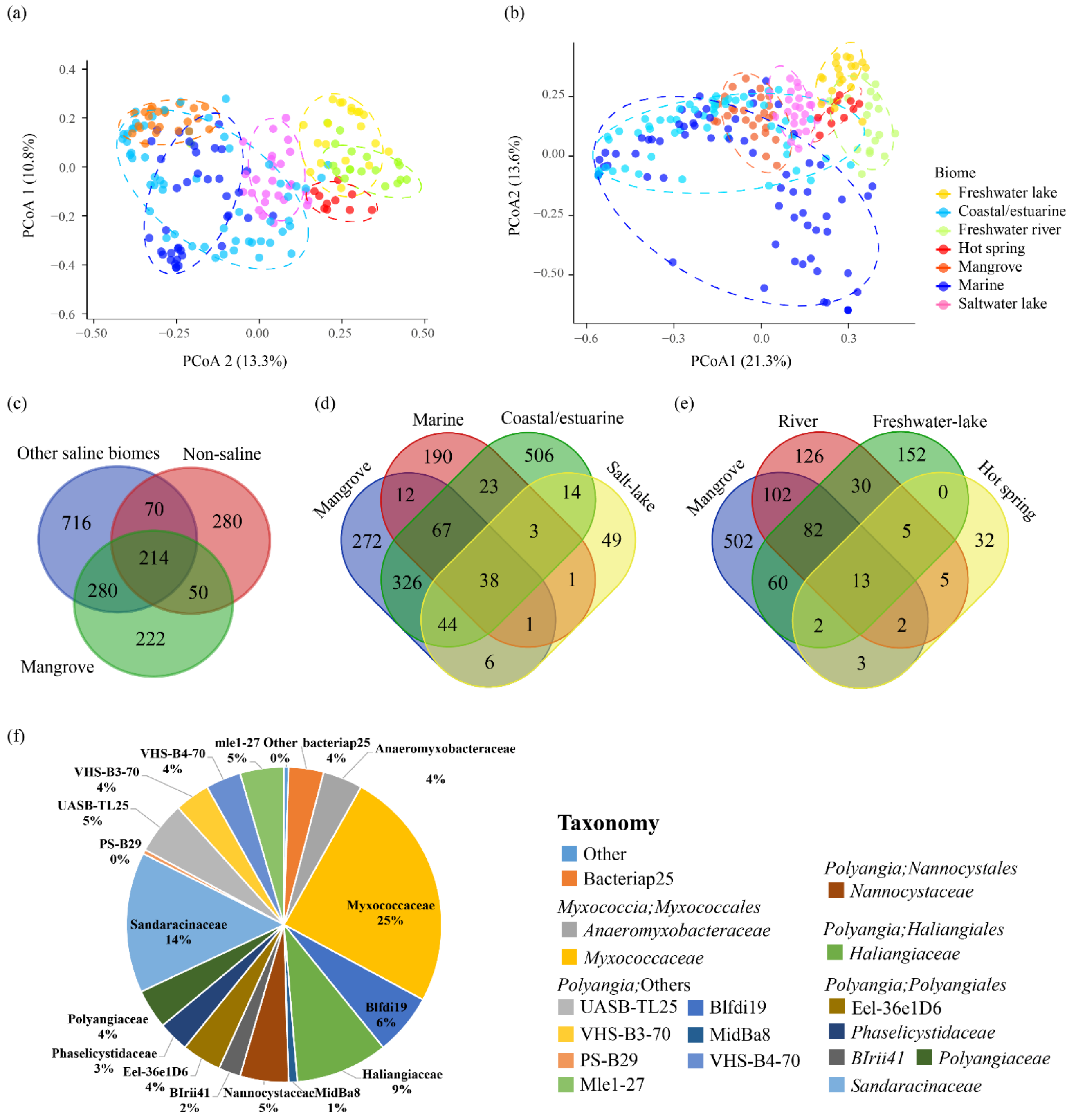

Figure S3 The results of PCoA for the total microbial community (a) and the *Myxococcota* community (b) in different biomes. The Venn figure (c) shows the shared and unique myxococcotal OTU numbers between mangrove, non-saline, and other saline environments. The venn figure (d) and (e) shows the source of mangrove myxococcotal OTUs from saline (marine, coastal, and salt-lake) biomes and non-saline (river, freshwater-lake, and hot spring) environments. The taxonomy profile of uniqe myxococcotal OTUs in the Venn figures in family-level (f).

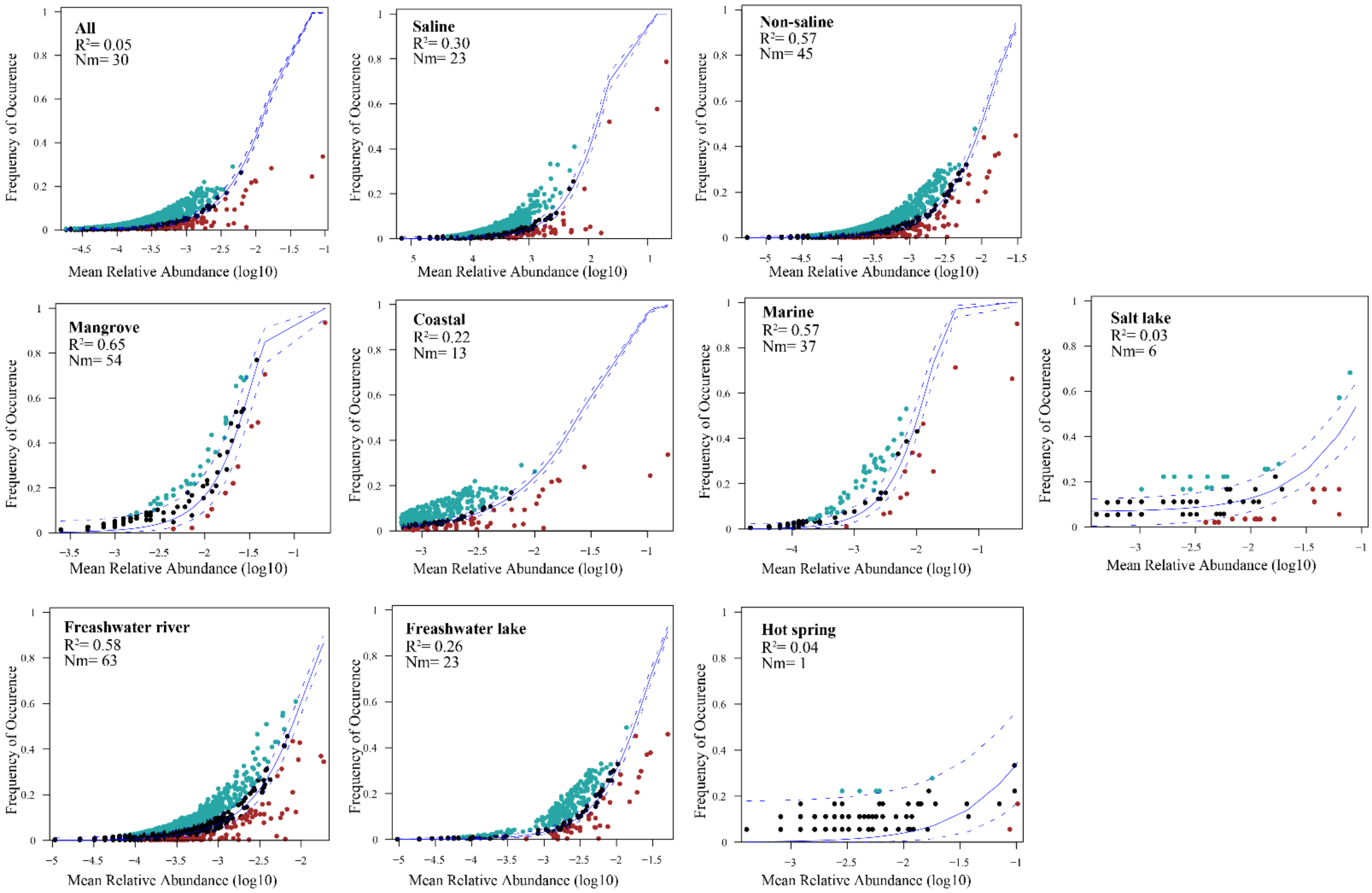

Figure S4 Fitting of the neutral community model (NCM) of *Myxococcota* community assembly in saline, non-saline, and individual habitats. Solid blue lines, the best fit to NCM. Dashed blue lines, 95% confidence interval for the predication. OTUs that occur more or less frequently than predicted by the NCM are denoted by different colors. Nm, the metacommunity size times immigration; R^2, fit to the model.

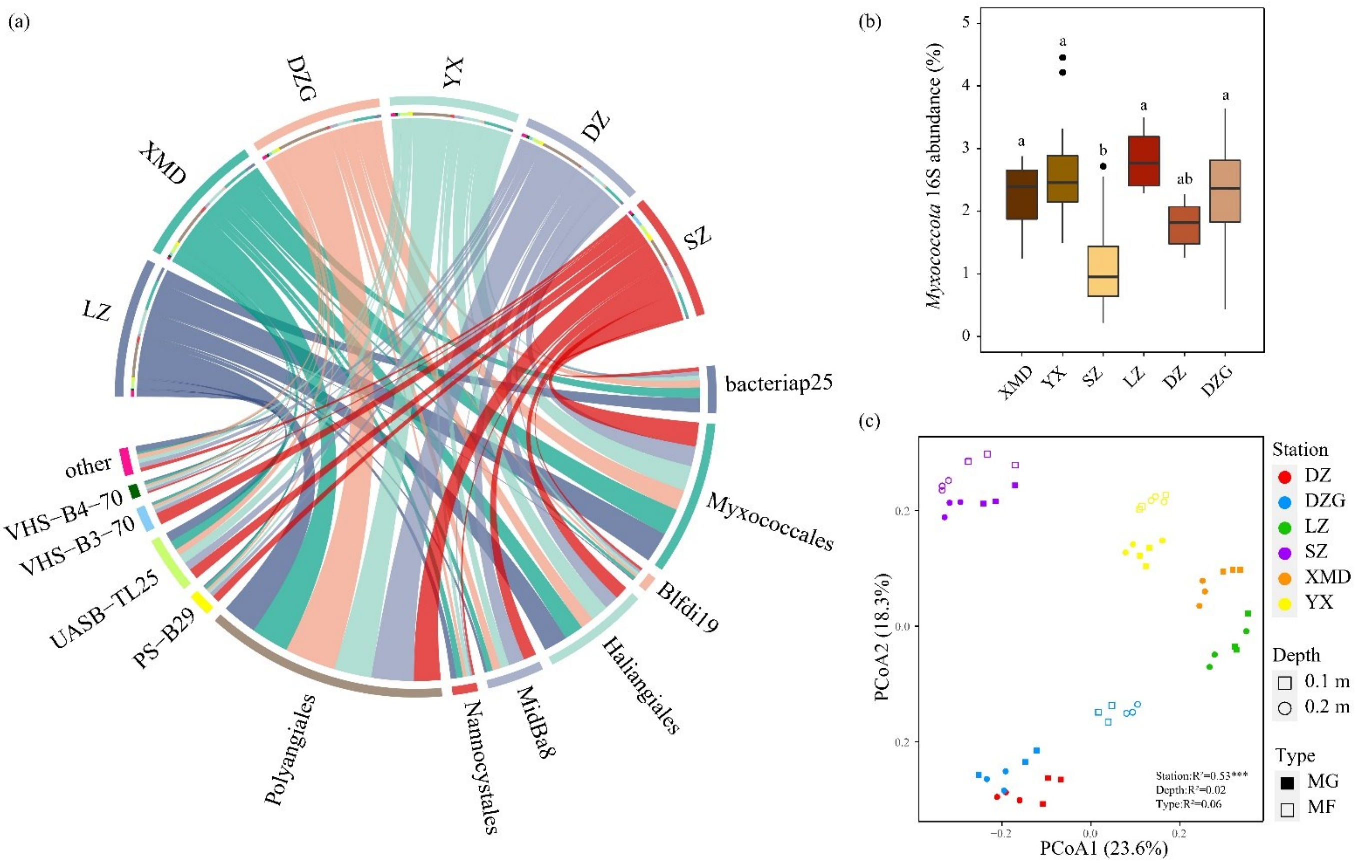

Figure S5 The community composition of *Myxococcota* in different mangroves (a). The colors of the top half of the outer ring represent mangroves located in LZ, XMD, DZG, YX, DZ, and SZ, respectively. The lower half of the outer ring is colored according to the major *Myxococcota* family. Line thickness corresponds to the relative abundance in different mangroves. The comparison of mangrove *Myxococcota* 16S gene abundance of samples collected in different mangroves (b). The PCoA result of *Myxococcota* community in different mangroves (c), based on the Bray-Curtis distance matrix. Similarity values among the samples of different mangroves ("Dtation"), depths ("Depth"), and sample type (i.e., mangrove or mudflat, "Type") were examined by using the analysis of similarities (ANOSIM) and are shown in the bottom right or left corner of this graph. ***: $p < 0.001$.

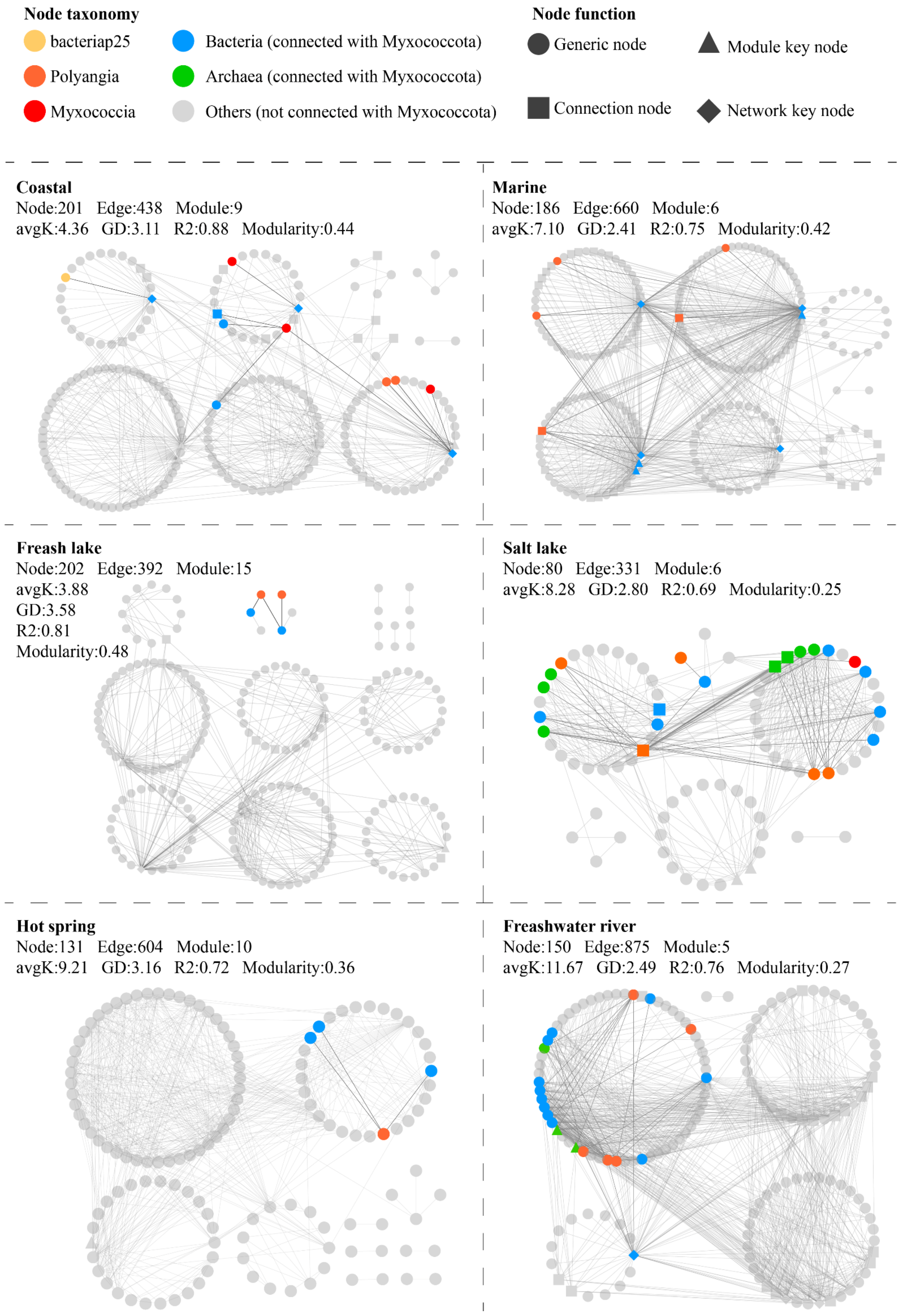
Node taxonomy
bacteriap25
Polyangia
Myxococcia
Bacteria (connected with Myxococcota)
Archaea (connected with Myxococcota)
Others (not connected with Myxococcota)
Node function
Generic node
Module key node
Connection node
Network key node
Coastal
Node:201 Edge:438 Module:9
avgK:4.36 GD:3.11 R2:0.88 Modularity:0.44
Marine
Node:186 Edge:660 Module:6
avgK:7.10 GD:2.41 R2:0.75 Modularity:0.42
Freash lake
Node:202 Edge:392 Module:15
avgK:3.88
GD:3.58
R2:0.81
Modularity:0.48
Salt lake
Node:80 Edge:331 Module:6
avgK:8.28 GD:2.80 R2:0.69 Modularity:0.25
Hot spring
Node:131 Edge:604 Module:10
avgK:9.21 GD:3.16 R2:0.72 Modularity:0.36
Freshwater river
Node:150 Edge:875 Module:5
avgK:11.67 GD:2.49 R2:0.76 Modularity:0.27

Figure S6 Microbial cooccurrence networks in different environments. Nodes belong to class bacteriap25, *Polyangia*, and *Myxococcia* of *Myxococcota* are colored by yellow, orange, and red, respectively. Other archaeal and bacterial nodes that are associated with *Myxococcota* nodes are colored by green and blue, respectively. Different shapes stand for different functions of nodes. avgK, average degree; GD, average path distance.
